# Clinical response to subcutaneous immunotherapy at 3 years in allergic rhinitis patients is predicted by short‐term treatment effectiveness

**DOI:** 10.1002/clt2.12223

**Published:** 2023-02-09

**Authors:** Dong Liu, Jingyun Li, Yunbo Gao, Feifei Cao, Wei Xiong, Chengshuo Wang, Yuan Zhang, Luo Zhang

**Affiliations:** ^1^ Department of Allergy Beijing Tongren Hospital Capital Medical University Beijing China; ^2^ Department of Otolaryngology Head and Neck Surgery Beijing Tongren Hospital Capital Medical University Beijing China; ^3^ Beijing Laboratory of Allergic Diseases and Beijing Key Laboratory of Nasal Diseases Beijing Institute of Otolaryngology Beijing China; ^4^ Research Unit of Diagnosis and Treatment of Chronic Nasal Diseases Chinese Academy of Medical Sciences Beijing China

To the Editor,

Allergic rhinitis (AR) is one of the most common airway diseases caused by specific immunoglobulin E (sIgE)‐mediated reactions against inhaled allergens and is clinically characterized by itching, sneezing, rhinorrhoea, and obstruction. Allergen immunotherapy (AIT) is considered an effective and safe aetiology‐oriented treatment for AR patients for whom symptom‐relieving therapy is ineffective, which is recommended to be continued up to 3 years to achieve better and long‐term efficacy.^1^ However, individual differences in the response to AIT are significant.^2^, ^3^, ^4^ Systematic reviews and meta‐analyses, considering treatment response as a primary outcome, showed considerable heterogeneity in the reported clinical response to AIT.^4^ Moreover, few studies compared the efficacy of short‐term and 3‐year AIT therapy to predict prognosis early in the treatment course.

We carried out a prospective observational study to analyze changes in the clinical response at different time points throughout AIT therapy and aimed to predict the final clinical response to AIT based on short‐term subjective efficacy. Participants with a confirmed diagnosis of AR and a major allergen of house dust mite (HDM) were recruited from the Department of Otolaryngology Head and Neck Surgery and Department of Allergy, Beijing Tongren Hospital between July 2017 and July 2019 and accepted standard subcutaneous immunotherapy (SCIT) with *Dermatophagoidespteronyssinus* (*Der p*) (Alutard SQ, ALK, Hørsholm, Denmark). The response to AIT was calculated as changes in average total combined score (ATCS) between baseline and 3‐year (end of treatment) visit (ATCSΔ3y)/baseline ATCS. Patients were divided into high responders and low/non responders according to the response to AIT. Details on the methods and materials are provided as Supplementary information S1 ‐ Additional file.

The study population was composed of 61 patients. The demographic characteristics of enrolled patients at baseline are presented in Table S1. At the end of 3 years of treatment, 35 patients (57.4%) exhibited high response, and 26 (42.6%) exhibited low/non response, indicating that most patients benefitted from HDM SCIT. The two groups were comparable with respect to age, sex, AR duration, co‐morbidity of asthma, history of smoking and drinking, baseline symptom scores and levels of sIgE in serum. Compared to the low/non responders, the high responders had more frequent sensitization to pure HDM (percentage: 68.6% vs. 50%) and higher ATCS at baseline (median: 10 vs. 8), albeit not significantly.

Standard subcutaneous immunotherapy significantly reduced individual AR symptom scores, total nasal symptom score (TNSS) and ATCS as early as 8 weeks after the initiation of treatment, except the score of sneezing (Figure 1A). The improvement in ATCS between baseline and each visit time point were defined as ATCSΔ8w, ATCSΔ6m, ATCSΔ1y, ATCSΔ2y, and ATCSΔ3y, respectively. ATCSΔ3y were significantly correlated with ATCSΔ8w (*r* = 0.39, *p* < 0.01), ATCSΔ6m (*r* = 0.45, *p* < 0.001), ATCSΔ1y (*r* = 0.41, *p* < 0.001) and ATCSΔ2y (*r* = 0.48, *p* < 0.0001) (Figure S1).

**FIGURE 1:**
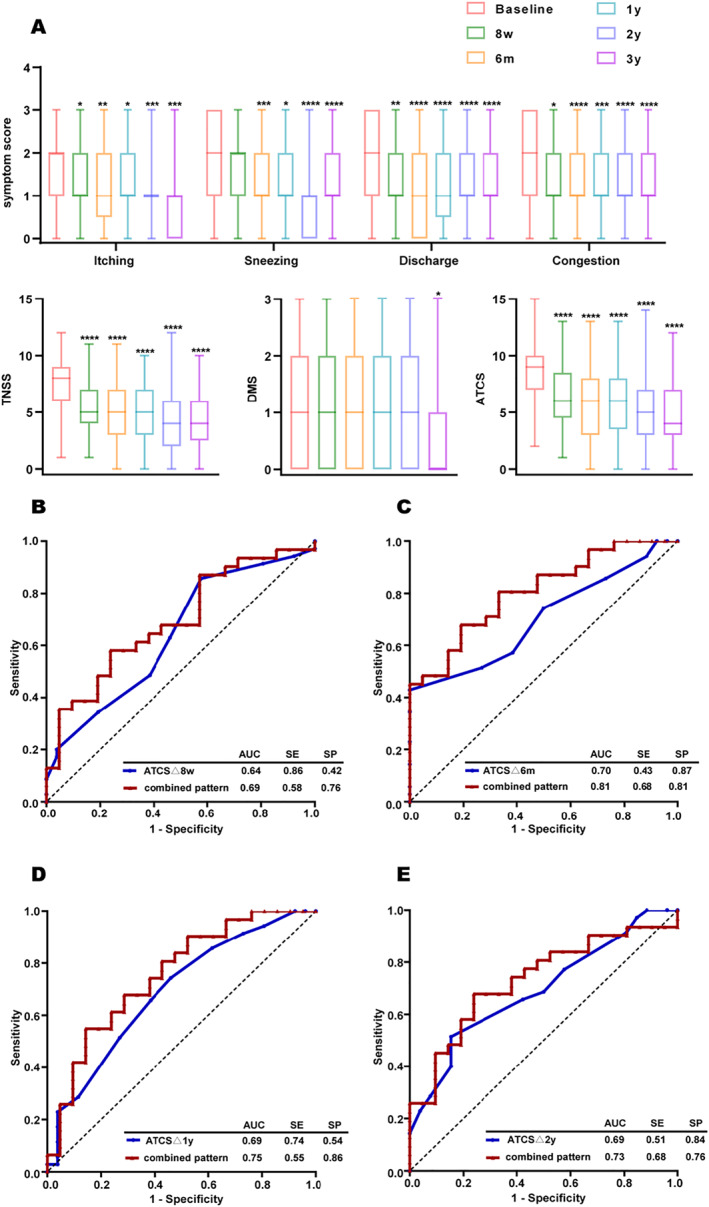
Evalution of short‐term treatment effectiveness in predicting clinical response to SCIT at 3 years. (A) Comparison of symptom scores, total nasal symptom score (TNSS), daily medication score (DMS), and average total combined score (ATCS), at each visit time point (8‐week, 6‐month, 1‐year, 2‐year and 3‐year). (B–E) Receiver operating characteristic (ROC) curves of improvement in ATCS between baseline and four visit time points (8‐week, 6‐month, 1‐year and 2‐year) or combined with house dust mite (HDM) sIgE to *Der p*, *Der f*, and *phadiatop* at baseline (combined pattern) for SCIT patients with high *vs*. low/non responese. *p < 0.05; **p < 0.01; ***p < 0.001; ****p < 0.0001. ATCS, average total combined score (TNSS + DMS); DMS, daily medication score; TNSS, total nasal symptom score; AUC, area under the curve; ROC, receiver operating characteristic; SCIT, subcutaneous immunotherapy

In our study, the differences in ATCS improvements between high response and low/non response groups were statistically significant (*p* < 0.05) from 6 months until the end of treatment (Figure S2). As investigated by receiver operating characteristic analyses, ATCSΔ6m (area under the curve [AUC] = 0.70, *p* = 0.007), ATCSΔ1y (AUC = 0.69, *p* = 0.012), and ATCSΔ2y (AUC = 0.69, *p* = 0.01) significantly predicted the clinical response to SCIT at 3 years (Figure 1B–E and Table S2). Furthermore, we combined ATCSΔ with biomarker levels (HDM sIgE to *Der p*, *Der f*, and *phadiatop* at baseline) in a composite model, which did indeed improve the diagnostic power over ATCSΔ alone (Table S2).

This prospective observational study revealed a correlation between short‐term treatment effectiveness and clinical response to 3‐year SCIT. In terms of symptom improvement, high responders and low/non responders showed a significant difference after 6 months of therapy. Further, we found that short‐term treatment effectiveness could discriminate the clinical response to AIT, and when combined with the ratio of sIgE to total IgE (sIgE/tIgE), the diagnostic power was improved.

Although the efficacy of AIT is widely accepted, not all patients benefit from AIT. Our observations suggest the majority of patients (57.4%) exhibited high response and the other (42.6%) exhibited low/non response, which confirm the efficacy of SCIT with HDM. In line with our study, Zimmer, et al.^5^ and Bordas‐Le Floch, et al.^6^ demonstrated more than half of patients received AIT revealed high response. We observed that the percentage of mono‐sensitized patients and the composite scores (TNSS and ATCS) at baseline tended to be higher (albeit not significantly) in high responders, which may contribute to AIT treatment outcomes.

Subsequently, our results showed an early effectiveness of HDM SCIT from 8 weeks after treatment initiation and a moderate correlation between primary outcome (ATCSΔ3y) and ATCS improvement at each visit time point, which indicate that short‐term effectiveness may be a predictor of the response of HDM SCIT. Differences in clinical response improvement between the two groups appeared after 6 months of SCIT and continued until the end of therapy. Receiver operating characteristic curve analysis indicated that improvement in ATCS in 6 months, 1 year, and 2 years may be predictive of the final clinical response to AIT. Therefore, close monitoring short‐term efficacy is recommended to identify clinical response to AIT treatment and guide individualized therapy. Di Lorenzo, et al.^7^ evaluated sIgE/tIgE as a predictive marker in AR patients who accepted HDM AIT for 4 years, using a sIgE/tIgE cut‐off value of 16.2. However, our findings revealed no difference in sIgE/tIgE between the two groups at baseline, but when combined with ATCS, it did improve the classification model. This discordance is likely because we used real‐world data, including from poly‐sensitized patients.

To our knowledge, this is the first study to comprehensively compare temporal changes in clinical response over the SCIT period between high‐ and low/non‐responder groups of AR patients and indicate that the clinical response to SCIT in AR can be predicted by early efficacy. We believe that these findings should help to understand temporal changes in clinical efficacy over the SCIT period.

## AUTHOR CONTRIBUTIONS

Study design: Dong Liu, Jingyun Li, Yuan Zhang. Medical expertise: Dong Liu, Jingyun Li, Yunbo Gao, Feifei Cao, Wei Xiong, Chengshuo Wang, Yuan Zhang, Luo Zhang. Statistical analysis: Jingyun Li, Yunbo Gao. Manuscript writing: All authors. Final approval: All authors.

## CONFLICT OF INTEREST

The authors declare that they have no competing interests.

## FUNDING INFORMATION

National key R&D program of China, 2022YFC2504100. The program for the Changjiang scholars and innovative research team, IRT13082. CAMS innovation fund for medical sciences, 2019‐I2M‐5‐022. Capital's funds for health improvement and research, 2022‐1‐1091. Natural Science Foundation of China, 81970849, 82071022 and 82271141. Beijing Municipal Science & Technology Commission, Z211100002921060.

## CONSENT FOR PUBLICATION

All the authors agree on publishing the submitted document.

## Supporting information

Supplementary Material

## Data Availability

The data that support the findings of this study are available from the corresponding author upon reasonable request.
