# Supplementary material for: Clinical response to subcutaneous immunotherapy at 3 years in allergic rhinitis patients is predicted by short‐term treatment effectiveness
Source: Clin Transl Allergy. 2023 Feb 9;13(2):e12223. doi: 10.1002/clt2.12223 (PMC9911623; doi:10.1002/clt2.12223)

**SUPPLEMENTARYMETHODS AND MATERIALS**

**Patients**

In this prospective observational study, a total of 86 participants with a confirmed diagnosis of AR according to the ARIA criteria^E1^ were recruited from the Department of Otolaryngology Head and Neck Surgery and Department of Allergy, Beijing Tongren Hospital. Subjects who showed significant nasal symptoms (rhinorrhoea, congestion, itching, and sneezing) were recruited from allergy outpatient clinic between July 2017 and July 2019 and accepted standard SCIT (Alutard SQ). All subjects were examined for sIgE using the Pharmacia UniCAP system (Thermo Fisher Scientific China Co., Ltd., Shanghai, China) and a commercial kit (Euroline Test^®^, Euroimmun, Lübeck, Germany). HDM was confirmed to be the major allergen based on a positive UniCAP test (sIgE ≥ 3.5 kAU/L) and was identified by a physician to be responsible for the most intense and/or bothersome symptoms.^E2^ Patients with severe unstable disease, including uncontrolled asthma and uncontrolled active systemic/immunologic disease, and pregnant or lactating women were excluded.

**Study design**

Eligible participants received 3 years of SCIT with Alutard^®^ SQ *Dermatophagoidespteronyssinus* (*Der p*) (ALK, Hørsholm, Denmark), and their demographic and clinical data were collected. According to the standard cluster immunotherapy schedule,^E3^ the treatment consisted of 8 weeks of up-dosing (up to 100,000 SQ-U) and 3-year maintenance with injection intervals of 6 ± 2 weeks.

At baseline of the study, serum total IgE (tIgE) and HDMsIgE levels to *Der p*, *Dermatophagoides farina* (*Der f*), and*phadiatop* (*phad*) were determined using UniCAP, following the manufacturer’s instructions.

The study was proved by the Medical Ethics Committee of Beijing Tongren Hospital. Written informed consent was obtained from all participants and guardians of minors prior to performing any procedures.

**Clinical efficacy**

The primary clinical objective was to assess the efficacy during the 3-year treatment. Efficacy was quantified using the total nasal symptom score (TNSS), daily medication score (DMS), and average total combined score (ATCS). ^E4, E5^ TNSS was the sum of scores of nasal symptoms of congestion, rhinorrhoea, sneezing, and itching, which were scored according to symptom severity on a four-point scale (0 = no symptoms, 1 = mild symptoms, 2 = moderate symptoms, 3 = severe symptoms). Thus, TNSS ranged from 0 to 12. The DMS was calculated considering only the strongest medication (scale: 0–3; 0 = no treatment, 1 = taking antihistamines, 2 = taking intranasal corticosteroids, 3 = taking oral corticosteroids). The ATCS was calculated as the sum of TNSSand DMS. ^E5^

Scores were calculated at baseline, 8-week, 6-month, 1-year, 2-year, and 3-year (end of treatment) visits. Changes in ATCS between baseline and each visit time point were defined as ATCSΔ8w, ATCSΔ6m, ATCSΔ1y, ATCSΔ2y, and ATCSΔ3y, respectively. The response to AIT was calculated as ATCSΔ3y/baseline ATCS. Patients were divided into high responders and low/non responders according to the response to AIT, with a <40% decrease defined as low/non responders and a ≥40% decrease as high responders. ^E6, E7^

**Statistical analysis**

The principal statistical software used was SPSS (version 24.0; IBM, Armonk, NY, USA). Non-normally distributed data were expressed as medians and ranges. For non-parametric variables, the Mann–Whitney *U* test was used. Categorical data were analysed using Pearson’s chi-square test or Fisher’s exact test. The Wilcoxon rank sum test was used to analyse differences in clinical variables over time. For correlation analysis, we used non-parametric Spearman’s correlation. Receiver operating characteristic (ROC) curves and areas under the curves (AUCs) were used to evaluate the discriminative ability of short-term treatment effectiveness in discriminating clinical responses to SCIT at 3 years and evaluate its sensitivity and specificity. Logistic regression analyses were used to establish the diagnostic panel. Model fitness was determined using the Hosmer–Lemeshow statistic. *P*< 0.05 was considered to indicate statistical significance.

**Supplementary references**

E1. Bousquet J, Schünemann HJ, Togias A, et al. Next-generation Allergic Rhinitis and Its Impact on Asthma (ARIA) guidelines for allergic rhinitis based on Grading of Recommendations Assessment, Development and Evaluation (GRADE) and real-world evidence. *J Allergy Clin Immunol*. 2020;145(1):70-808.e3.

E2. Demoly P, Passalacqua G, Pfaar O, Sastre J, Wahn U. Management of the polyallergic patient with allergy immunotherapy: a practice-based approach. *Allergy Asthma Clin Immunol*. 2016;12:2.

E3. Zhang L, Wang C, Han D, Wang X, Zhao Y, Liu J. Comparative study of cluster and conventional immunotherapy schedules with dermatophagoidespteronyssinusin the treatment of persistent allergic rhinitis. *Int Arch Allergy Immunol*. 2009;148(2):161-169.

E4. Calderon MA, Bernstein DI, Blaiss M, Andersen JS, Nolte H. A comparative analysis of symptom and medication scoring methods used in clinical trials of sublingual immunotherapy for seasonal allergic rhinitis. *Clin Exp Allergy*. 2014;44(10):1228-1239.

E5. Demoly P, Corren J, Creticos P, et al. A 300 IR sublingual tablet is an effective, safe treatment for house dust mite-induced allergic rhinitis: An international, double-blind, placebo-controlled, randomized phase III clinical trial. *J Allergy Clin Immunol*. 2021;147(3):1020-1030.e10.

E6. Zimmer A, Bouley J, Le Mignon M, et al. A regulatory dendritic cell signature correlates with the clinical efficacy of allergen-specific sublingual immunotherapy. *J Allergy Clin Immunol*. 2012;129(4):1020-1030.

E7. Bordas-Le Floch V, Berjont N, Batard T, et al. Coordinated IgG2 and IgE responses as a marker of allergen immunotherapy efficacy. *Allergy*. 2022;77(4):1263-1273.

**SUPPLEMENTARYTABLES**

**Table S1** Baseline characteristics and response to SCIT at 3 years in the cohort.

| Characteristic | SCIT patients  (n = 61) | Response | | |
| --- | --- | --- | --- | --- |
|  |  | High responders  (n = 35) | Low/non responders  (n = 26) | *P*^#^ |
| Age (years), median (Q1, Q3) | 31.0 (16.0, 37.0) | 30.0 (16.0, 39.0) | 33.5 (19.5, 36.0) | 0.86 |
| Children, n (%) | 10 (16.4) | 6 (17.1) | 4 (15.4) | 0.85 |
| Gender: male, n (%) | 38 (62.3) | 20 (57.1) | 18 (69.2) | 0.34 |
| AR duration (years), (Q1, Q3) | 5.0 (5.0, 10.0) | 5.0 (4.3, 8.8) | 6.5 (5.0, 10.0) | 0.34 |
| Asthma, n (%) | 7 (11.5) | 4 (11.4) | 3 (11.5) | 1.00 |
| Pure HDM sensitization, n (%) | 37 (60.7) | 24 (68.6) | 13 (50.0) | 0.14 |
| Smoking, n (%) | 7 (11.5) | 4 (11.4) | 3 (11.5) | 1.00 |
| Drinking, n (%) | 7 (11.5) | 3 (8.6) | 4 (15.4) | 0.20 |
| Symptom Score, median (Q1, Q3) | | | | |
| Itching | 2.0 (1.0, 2.0) | 2.0 (1.0, 3.0) | 2.0 (1.0, 2.0) | 0.13 |
| Sneezing | 2.0 (1.0, 3.0) | 2.0 (1.5, 3.0) | 2.0 (1.0, 3.0) | 0.42 |
| Discharge | 2.0 (1.0, 3.0) | 2.0 (1.5, 3.0) | 2.0 (1.3, 2.0) | 0.55 |
| Congestion | 2.0 (1.0, 3.0) | 2.0 (1.5, 2.5) | 2.0 (1.3, 2.8) | 0.83 |
| TNSS, median (Q1, Q3) | 8.0 (6.0, 9.0) | 8.0 (7.0, 9.5) | 7.5 (5.3, 9.0) | 0.23 |
| DMS, median (Q1, Q3) | 1.0 (0.0, 2.0) | 2.0 (0.0, 2.0) | 0.5 (0.0, 2.0) | 0.16 |
| ATCS, median (Q1, Q3) | 9.0 (7.0, 10.0) | 10.0 (7.0, 10.5) | 8.0 (6.3, 9.0) | 0.063 |
| Serum sIgE to *Der p* (kAU/L), median (Q1, Q3) | 3.3 (1.0, 17.2) | 3.2 (1.0, 14.0) | 3.3 (1.1, 20.3) | 0.54 |
| Serum sIgE to *Der f* (kAU/L), median (Q1, Q3) | 6.2 (1.3, 21.6) | 5.8 (1.2, 21.4) | 6.2 (3.8, 27.6) | 0.43 |
| Serum sIgE to *phad* (kAU/L), median (Q1, Q3) | 4.2 (1.2, 20.4) | 5.4 (1.1, 21.3) | 3.72 (1.9, 19.6) | 0.66 |
| Serum total IgE (kU/L), median (Q1, Q3) | 185.0 (74.9, 557.0) | 183.0 (91.6, 488.5) | 308.5 (67.8, 757.8) | 0.69 |

^#^Mann–Whitney*U* test was used for continuous variables, which are presented as median (interquartile range). Pearson’s chi-squared or Fisher’s exact test was used for categorical variables, which are presented as number (percentage). No significant differences were observed between high and low/non responders. *Abbreviations:* AR, allergic rhinitis; ATCS, average total combined score (TNSS+DMS); *Der f*, *Dermatophagoides farina*; *Der p*, *Dermatophagoides pteronyssinus*; DMS, daily medication score; HDM, house dust mite; *phad*, *phadiatop*; sIgE, specific immunoglobulin E; TNSS, total nasal symptom score.

**Table S2** Prediction of response to SCIT at 3 years using ROC analyses.

| Variable | ROC | | | Hosmer–Lemeshow test | |
| --- | --- | --- | --- | --- | --- |
|  | AUC | AUC 95CI% | *P* | χ^2^ | *P* |
| ATCSΔ8w | 0.64 | 0.50–0.78 | 0.063 | 5.67 | 0.34 |
| ATCSΔ6m | 0.70 | 0.57–0.83 | **0.007** | 12.15 | 0.059 |
| ATCSΔ1y | 0.69 | 0.55–0.82 | **0.012** | 0.23 | 1.00 |
| ATCSΔ2y | 0.69 | 0.56–0.82 | **0.010** | 7.24 | 0.51 |
| Combined pattern |  |  |  |  |  |
| ATCSΔ8w (*Der p*, *Der f*, and *phad*) sIgE/tIgE | 0.69 | 0.55–0.84 | **0.019** | 8.45 | 0.39 |
| ATCSΔ6m (*Der p*, *Der f*, and *phad*) sIgE/tIgE | 0.81 | 0.69–0.92 | **<0.001** | 8.34 | 0.40 |
| ATCSΔ1y (Der p, Der f, and phad) sIgE/tIgE | 0.75 | 0.62–0.89 | **0.002** | 5.46 | 0.71 |
| ATCSΔ2y (*Der p*, *Der f*, and *phad*) sIgE/tIgE | 0.73 | 0.59–0.87 | **0.005** | 4.44 | 0.82 |

^a^Modelled probability of responses of participants; only significant variables contributing to panels are listed. Statistics were conducted on log-transformed values of variables. *Abbreviations:* AUC, area under the curve; ATCS, average total combined score (TNSS+DMS); *Der f*, *Dermatophagoidesfarina*; *Der p*, *Dermatophagoidespteronyssinus*; *phad*, *phadiatop*; ROC, receiver operating characteristic; sIgE, specific immunoglobulin E.

**SUPPLEMENTARYFIGURELEGENDS**

**Figure S1** Correlations of ATCSΔ8w (A), ATCSΔ6m (B), ATCSΔ1y (C), and ATCSΔ2y with primary outcome (ATCSΔ3y). The change in ATCS at each visit time point showed a positive correlation with primary outcome. **P*＜0.05; ***P*＜0.01; ****P*＜0.001; *****P*＜0.0001. *Abbreviation:* ATCS, average total combined score (TNSS+DMS).


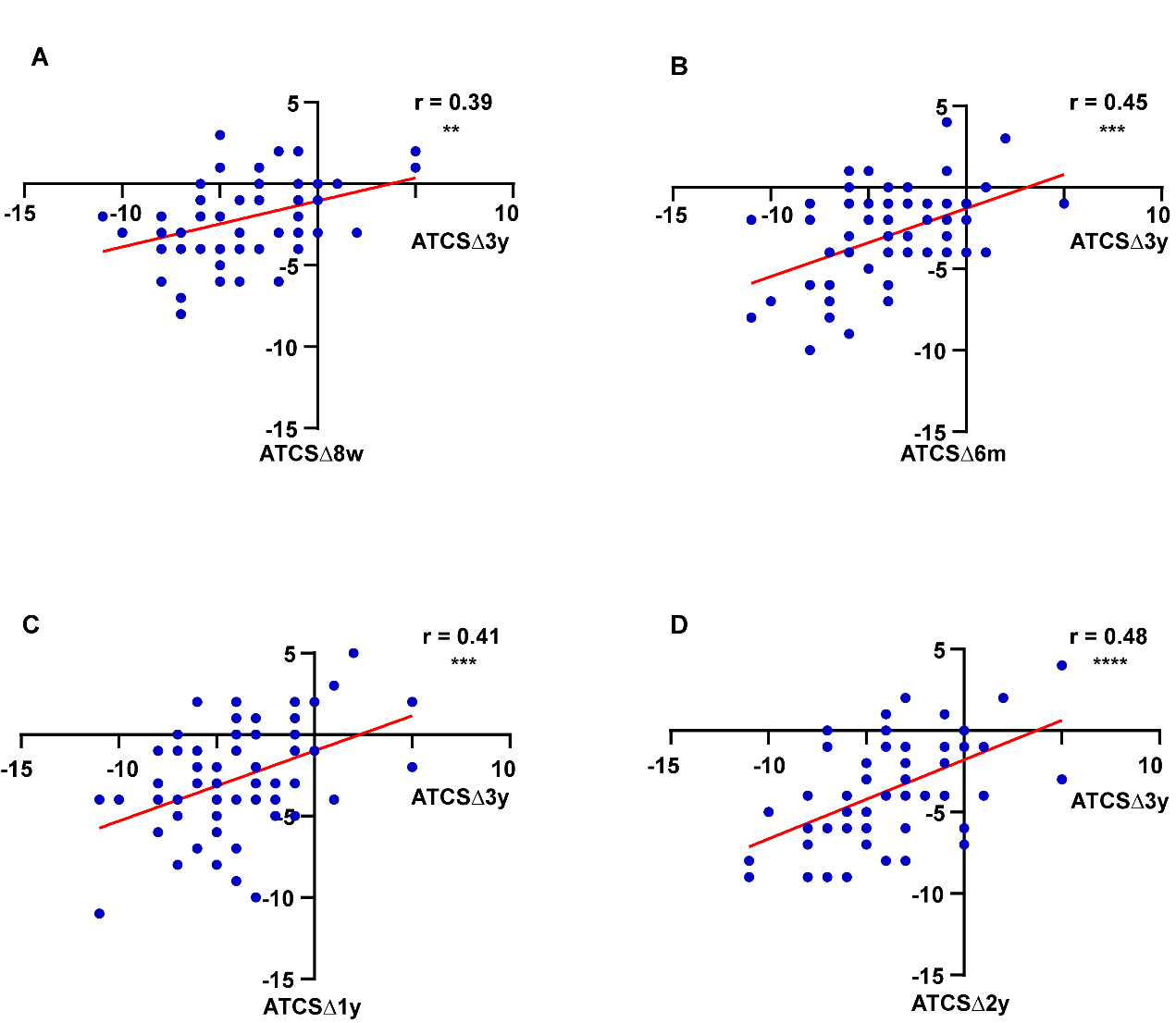


**Figure S2** Comparison of changes in ATCS at each visit time point to baseline between high and low/non responders.**P*＜0.05; ***P*＜0.01. *Abbreviations:* ATCS, average total combined score (TNSS+DMS).


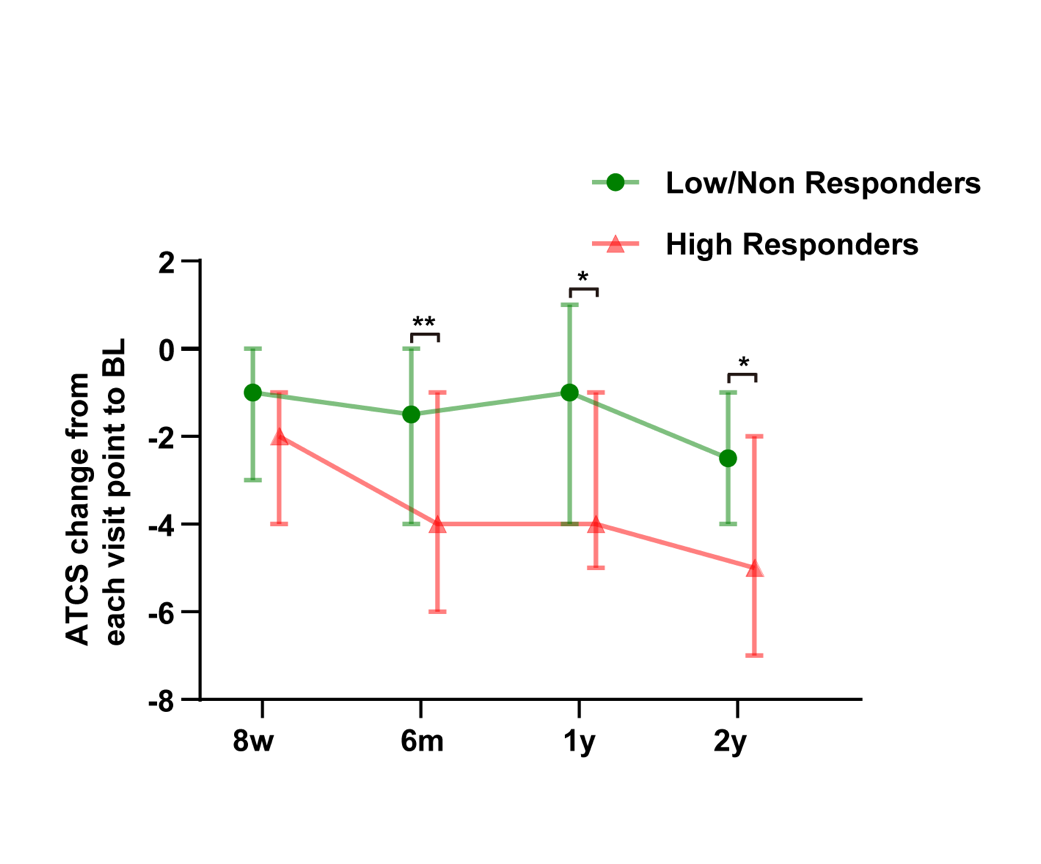

Supplement: Supplementary file 1 — Supplementary Material [file CLT2-13-e12223-s001.docx]
